# ASTROGLIA: Molecular Mechanisms, Functional Roles, and Neurophysiological Implications in the Central Nervous System

**DOI:** 10.3390/life15101505

**Published:** 2025-09-24

**Authors:** Andrea Ortega, Luz A. Martínez-Nuncio, Elisa Taddei, Eduardo Castañeda, Carmen Rubio, Moisés Rubio-Osornio

**Affiliations:** 1 Hospital General Manuel Gea González, Mexico City 14080, Mexico; andy@gmail.com; 2 Department of Neurochemistry, National Institute of Neurology and Neurosurgery MVS, Mexico City 14269, Mexico; alynn.nuncio@gmail.com; 3 Neurophysiology Laboratory, National Institute of Neurology and Neurosurgery MVS, Mexico City 14269, Mexico; elisataddey@gmail.com (E.T.); eduardocastaneda2510@gmail.com (E.C.)

**Keywords:** astroglia, neuroscience, synaptic homeostasis, neuronal metabolism, neuronal plasticity

## Abstract

Astrocytes, the principal components of astroglia, play essential roles in maintaining neuronal and synaptic homeostasis in the central nervous system. By regulating extracellular levels of glutamate, potassium (K^+^), and calcium (Ca^2+^), they prevent excitotoxicity and support neuronal survival. Astrocytes also modulate synaptic transmission and plasticity through gliotransmission, including vesicular glutamate release and D-serine synthesis via the serine shuttle, which regulates NMDA receptor activity. They provide metabolic support by facilitating glucose and oxygen transport from the vasculature, forming dynamic neurovascular units. Through signaling pathways such as cAMP-PKA and interactions with neurotrophic factors like BDNF and GDNF, astrocytes influence gene expression, synaptic remodeling, and plasticity. Furthermore, astrocytes exhibit regional and functional heterogeneity, which underlies their diverse contributions to both physiological and pathological conditions, including neurodegenerative diseases. This review summarizes current knowledge on astrocytic regulation of neuronal homeostasis, synaptic plasticity, and metabolism, highlighting their mechanisms of network communication, gliotransmission, and regional specialization, and discusses their implications in health and disease.

## 1. Introduction

Over the past three decades, astroglia, particularly astrocytes, have emerged as central regulators of brain function, moving beyond their traditional characterization as mere supportive elements in neurohistology [1]. The study of neuroglia began in the 19th century, with Otto Friedrich Karl Deiters (1834–1863) describing central nervous system cells lacking axons, initially termed “neurites” or “Deiters cells.” The term “neuroglia” was later coined by Rudolf Virchow in 1859, reflecting their assumed supportive role as “nerve glue.” While these historical milestones highlight the discovery of glia, the current focus is on the functional significance of astrocytes in CNS homeostasis and plasticity rather than an extended historical account. Astrocytes are now recognized as essential for maintaining neural homeostasis, modulating synaptic transmission, and supporting plasticity [2,3,4]. They regulate the extracellular environment by uptaking and recycling neurotransmitters, buffering ions such as potassium (K^+^) and calcium (Ca^2+^), and contributing to blood–brain barrier integrity. Astrocytes also facilitate energy metabolism by distributing glucose and lactate to neurons, particularly during high synaptic activity. As the principal macroglial cells of the C+NS, astrocytes are distinct from peripheral glia in origin and function [5]. They participate in specialized CNS processes, including synapse formation, maintenance of white matter, neurovascular coupling, and regulation of the tripartite synapse. Recent discoveries, such as astrocyte-neuron lactate shuttling, have emphasized their integral role in synaptic integration and information processing. Under pathological conditions, such as trauma, epilepsy, neurodegeneration, and psychiatric disorders, astrocytes undergo reactive astrogliosis, a complex transformation that may be protective or detrimental depending on the context and severity of the insult [6,7,8]. These changes involve alterations in morphology, cytoskeletal composition (including GFAP, vimentin, and desmin), calcium signaling, gliotransmitter release, and neurotrophic factor production. Cutting-edge single-cell transcriptomics and proteomics have revealed significant heterogeneity among astrocyte populations, with distinct subtypes exhibiting region-specific functions and differential responses to injury or disease. Understanding this heterogeneity is crucial for elucidating astrocytic contributions to CNS function and for identifying potential therapeutic targets. This review focuses on astrocytes and their central role in neural function, synaptic plasticity, and metabolic support, highlighting their molecular diversity, signaling mechanisms, and interactions with neurons, NG2 glial cells, oligodendroglia, and microglia. Sections have been reorganized to improve coherence, emphasizing astrocytic functions in health and disease, while peripheral glial cells are discussed only when relevant. We also address gliotransmission, neurovascular coupling, vesicular glutamate transport (including vGluts), and D-serine synthesis via the serine shuttle, highlighting their roles in NMDA receptor modulation and synaptic plasticity. Furthermore, astrocytic network communication is discussed in the context of Golgi’s syncytial concept versus Ramón y Cajal’s neuronal theory, showing how these perspectives may describe complementary mechanisms of CNS integration. This integrative review frames astrocytes as active regulators of the CNS, whose functional heterogeneity, signaling dynamics, and trophic interactions are critical for understanding both physiological processes and neurological disorders.

## 2. Peripheral Neuroglia: Context and Contrast

**Figure 1:**
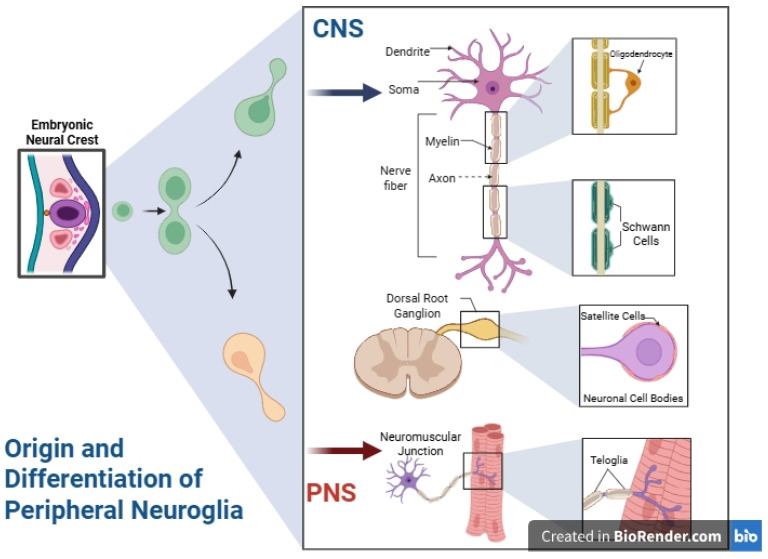
Origin and differentiation of peripheral neuroglia compared to central nervous system astroglia. During embryonic development, neural crest cells generate diverse peripheral glial populations Schwann cells, which ensheath peripheral axons (both myelinating and non-myelinating); satellite glial cells, which envelop neuronal cell bodies in dorsal root and sympathetic ganglia and contribute to microenvironment support and regulation; and terminal teloglia at neuromuscular junctions, which guide motor axon innervation and help maintain synaptic architecture. These peripheral glial types share overlapping characteristics, leading to the occasional classification of teloglia as a subtype of Schwann cells. In marked contrast, astrocytes in the central nervous system arise from distinct neuroepithelial origins and have roles tailored to the brain and spinal cord environment. This fundamental developmental and functional divergence underlies the exclusive focus of the present review on central astroglia. CNS, central nervous system; PNS, peripheral nervous system. Created in BioRender by Elisa Taddei, Luz A. Martinez Nuncio and Eduardo Castañeda. (2025) https://biorender.com/illustrations (accessed on 21 September 2025).

Although our review primarily focuses on astroglia within the central nervous system (CNS), we briefly contextualize peripheral glial cells to highlight the unique roles of astrocytes. Peripheral neuroglia comprises Schwann cells, satellite cells of somatic and visceral ganglia, and terminal glial cells or teloglia, all derived from the embryonic neural crest [9,10]. Among these, Schwann cells are the most extensively characterized; they ensheathe peripheral axons and generate the myelin sheath critical for rapid signal conduction [11,12,13]. Each axon and its glial covering constitute a nerve fiber, whether myelinated or unmyelinated [13,14]. Satellite cells in dorsal root and sympathetic ganglia surround neuronal bodies, mediating metabolic exchange with surrounding vasculature [15,16]. Terminal glial cells at neuromuscular junctions (NMJs) are integral to motor axon guidance and synaptic maintenance [17,18,19]. These peripheral glial types share some characteristics with Schwann cells but are functionally distinct from central astrocytes [20,21]. Similar glial elements are also present in autonomic plexuses, insulating neurons and axons in both enteric and visceral systems [22,23]. This summary underscores structural and functional differences between peripheral glia and astrocytes, emphasizing the latter’s central role in CNS homeostasis. The following sections focus exclusively on astrocytes, detailing their contribution to neuronal network organization, metabolic support, and synaptic plasticity (Figure 1).

## 3. Central Neuroglia: Historical Background and Taxonomy

Central neuroglia comprises neuroectoderm- and mesoderm-derived cells in the CNS, including astrocytes and diverse non-astrocytic glial types essential for neural function and homeostasis [24]. This section details non-astrocytic central glia, complementing peripheral glia discussion. Key types include oligodendrocytes, ependymal cells, NG2 glia (polydendrocytes/oligodendrocyte precursor cells), and specialized populations such as radial glia and tanycytes, each with distinct morphologies, molecular profiles, developmental origins, and functions [25]. Oligodendrocytes, derived from neuroectoderm, myelinate multiple CNS axons, supporting rapid conduction and axonal integrity. They also metabolically interact with neurons, supplying lactate and other substrates, especially in white matter [26,27]. Ependymal cells form the ventricular lining, regulate cerebrospinal fluid (CSF) homeostasis, contribute to CSF circulation via ciliary movement, and selectively mediate fluid exchange between CSF and parenchyma. Some, like subventricular ependymocytes, may participate in adult neurogenesis [28]. NG2 glia are widely distributed in gray and white matter, retaining proliferative capacity and potential differentiation into oligodendrocytes or reactive glia. They actively participate in synaptic signaling and neuroregenerative processes. Radial glia guides neuronal migration and may generate multiple glial lineages; tanycytes, lining the third ventricle, link CSF composition to hypothalamic circuits and nutrient sensing [25,29]. Collectively, non-astrocytic glia form a dynamic network interacting with neurons, vasculature, immune elements, and extracellular matrix, essential for CNS physiology and pathology [30,31,32].

### 3.1. Ependymal and Non-Astrocytic Central Glia: Anatomical Significance

**Figure 2:**
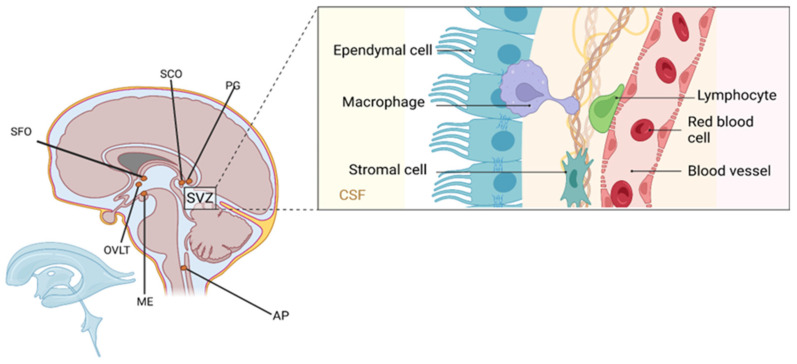
Anatomical organization of the subventricular zone (SVZ) and associated periventricular structures. The inset illustrates cellular components along the ventricular wall, including ependymal cells lining the cerebrospinal fluid (CSF)-filled lumen, underlying stromal cells, perivascular macrophages, lymphocytes, and red blood cells within adjacent blood vessels. These elements contribute to the composition of the diffuse periventricular system, which encompasses the subfornical organ (SFO), organum vasculosum of the lamina terminalis (OVLT), subcommissural organ (SCO), pineal gland (PG), area postrema (AP), and median eminence (ME). Ependymal cells, while not astrocytes, play critical structural and secretory roles and serve as an interface between the brain parenchyma and CSF, highlighting the anatomical and functional complexity of non-astrocytic glia in CNS homeostasis. Created in BioRender by Elisa Taddei, Luz A. Martinez Nuncio and Eduardo Castañeda. (2025) https://biorender.com/illustrations (accessed on 21 September 2025).

Ependymal cells contribute to CNS design and homeostasis. Santiago Ramón y Cajal, though omitting them from early neuroglial classification, described their morphology in detail [28]. They delineate ventricular systems and develop specialized variants, including choroid plexus epithelium for CSF synthesis [33,34]. Basal third ventricle ependymal cells form periventricular organs (vascular organ of lamina terminalis, subfornical organ, pineal gland, area postrema, etc.) [35,36] (Figure 2). Certain circumventricular organs also contain specialized astrocytes, highlighting astroglia’s integrative neuroendocrine roles [37,38]. Although non-astrocytic glia has distinct roles, astrocytes’ diversity, plasticity, and signaling capabilities position them as primary CNS regulators, the central theme of this review.

### 3.2. Oligodendroglia and Microglia: Non-Astrocytic Central Nervous System Glia

**Figure 3:**
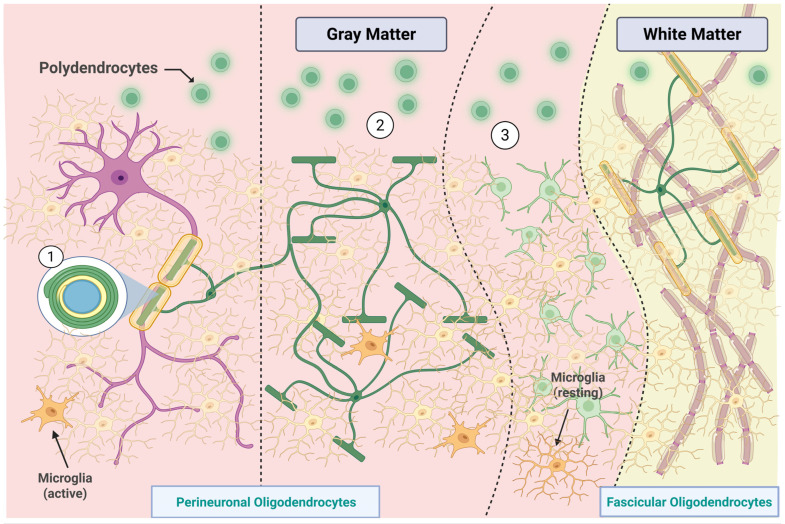
Distribution and classification of oligodendroglial and microglial subtypes in gray and white matter. In the gray matter, perineuronal oligodendrocytes are categorized based on their spatial relationship with neurons: (1) direct contact, (2) intermediate association, and (3) distant positioning with myelinating functions. Polydendrocytes (or oligodendrocyte progenitor cells) are shown in proximity to both neuronal and vascular structures, reflecting their role in remyelination and plasticity. Microglia, the smallest CNS glial cells, appear in both active and resting states, depending on their local microenvironment. In white matter, fascicular oligodendrocytes extend multiple processes to generate myelin sheaths, facilitating high-velocity conduction. This spatial and morphological heterogeneity underscores the specialized contributions of non-astrocytic glial cells to central nervous system structure and function. Created in BioRender by Elisa Taddei, Luz A. Martinez Nuncio and Eduardo Castañeda. (2025) https://biorender.com/illustrations (accessed on 21 September 2025).

Oligodendrocytes, including perineuronal/satellite and fascicular types, myelinate multiple axons contrasting with Schwann cells that typically myelinate single axon segments. NG2 glia, a highly distributed progenitor population, participates in remyelination and plasticity [25,39]. Microglia, mesoderm-derived CNS immune cells (5–7 μm), continuously surveil the brain. Upon injury or infection, they become phagocytic, clearing debris and modulating inflammation [40,41,42,43]. While this review focuses on astrocytes, these comparisons contextualize their specialization relative to oligodendrocytes and microglia (Figure 3).

## 4. Astrocytes: Glycogen Metabolism, Cytoskeletal Characteristics, and Blood–Brain Barrier Function

**Figure 4:**
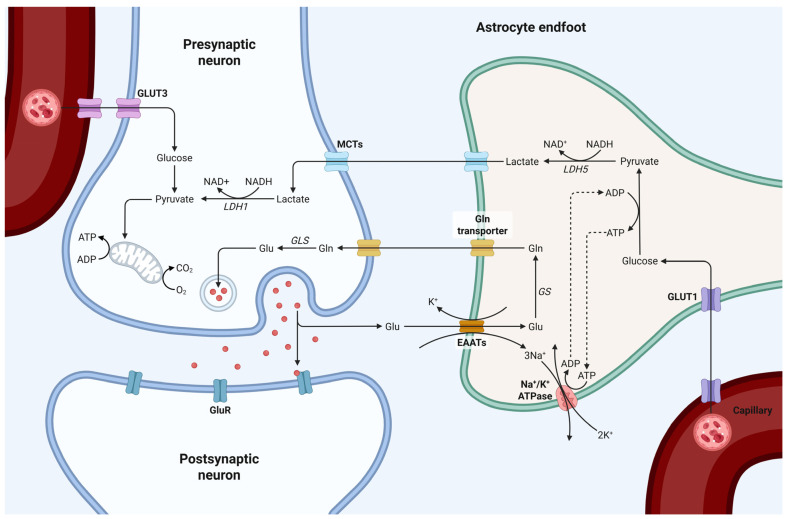
Schematic representation of astrocyte-neuron metabolic coupling at the synapse. Glu-cose is transported from the bloodstream into astrocytes via GLUT1, where it undergoes glycolysis and is converted to lactate, by lactate dehydrogenase 5 (LDH5). Lactate is then transported to neurons through monocarboxylate transporters (MCTs), where it is converted back to pyruvate by LDH1 and enters the mitochondrial TCA cycle for ATP production. Glutamate released from the presynaptic neuron is taken up by astrocytes via excitatory amino acid transporters (EAATs), converted to glutamine, by glutamine synthetase (GS), and shuttled back to neurons to replenish neurotransmitter pools via glutaminase (GLS). This tightly regulated metabolic interaction highlights the essential role of astrocytes in maintaining synaptic function, neurotransmitter recycling, and energy support. Created in BioRender by Elisa Taddei, Luz A. Martinez Nuncio and Eduardo Castañeda. (2025) https://biorender.com/illustrations (accessed on 21 September 2025).

Astrocytes are crucial for sustaining energy homeostasis in the CNS by means of glycogen storage and metabolism. They inherently accumulate glycogen and exhibit enzymes essential for glycogenolysis. Glucose is metabolized to pyruvate and subsequently lactate, which serves as an energy substrate for neurons via the astrocyte-neuron lactate shuttle. At non-myelinated axonal junctions, lactate is converted into pyruvate, which then enters the mitochondrial tricarboxylic acid (Krebs) cycle, undergoing oxidative metabolism to generate ATP essential for maintaining nerve conduction, synaptic activity, and neurotransmitter cycling [44] (Figure 4).

Astrocytes regulate the extracellular ionic composition through mechanisms reliant on electrochemical gradients. Ions such as Na^+^, K^+^, Ca^2+^, and Cl^−^ diffuse rapidly in the limited extracellular space of the CNS, driven by concentration and electrical gradients [45,46]. Astrocytic membranes possess ion channels and transporters that respond to voltage fluctuations, ligand interactions, and phosphorylation, including Kir4.1, two-pore domain potassium channels, and Kv10.1, enabling precise ion homeostasis [47]. These functions are essential for preserving synaptic transmission, preventing excitotoxicity, and supporting network excitability.

A fundamental anatomical characteristic of astrocytes is their role in the blood–brain barrier (BBB). Astrocytic endfeet, specifically the processes contacting blood vessels, envelop cerebral microvessels, maintaining BBB integrity and modulating molecular exchange between the vasculature and neural tissue. Other astrocytic processes contact neurons and synapses, enabling gliotransmission and metabolic support. This structural support is mediated by the astrocytic cytoskeleton, which consists of gliofilaments derived from glial fibrillary acidic protein (GFAP), as well as microtubules and actin microfilaments [48]. GFAP forms the core of intermediate filaments, providing mechanical stability and facilitating endfoot anchoring, astrocytic process remodeling, and synaptic plasticity [49]. GFAP comprises multiple isoforms (α, β, μ, Δ/k, Δ135, Δ164, Δexon6) with differential expression across astrocyte subtypes, including radial glia, Müller cells, Bergmann glia, tanycytes, and some peripheral glia [50,51,52,53]. GFAPΔ, is associated with astrocytic stem potential in subventricular and subpial zones. Astrocytes actively release thrombospondins (TSPs), which promote synaptogenesis, although the resulting synapses may be initially “silent,” requiring additional astrocytic signals for full functional maturation. These processes are enriched in gray matter astrocytes and are particularly critical during development and experience-dependent synaptic plasticity [54,55]. Importantly, astrocytes participate in gliotransmission by releasing glutamate, ATP, and D-serine. Vesicular glutamate transporters (vGluts) in astrocytes enable regulated glutamate release, contributing to synaptic modulation and tripartite synapse function as described by Araque et al. (2001–2019). Additionally, astrocytes synthesize D-serine via the serine shuttle, modulating NMDA receptor activity and influencing synaptic plasticity. Astrocytic network communication also supports the concept of CNS syncytial organization, complementing Ramón y Cajal’s neuronal theory, and allowing integrated modulation of excitability across neuronal populations.

## 5. Fibrous Astrocytes: Anatomy and Role

**Figure 5:**
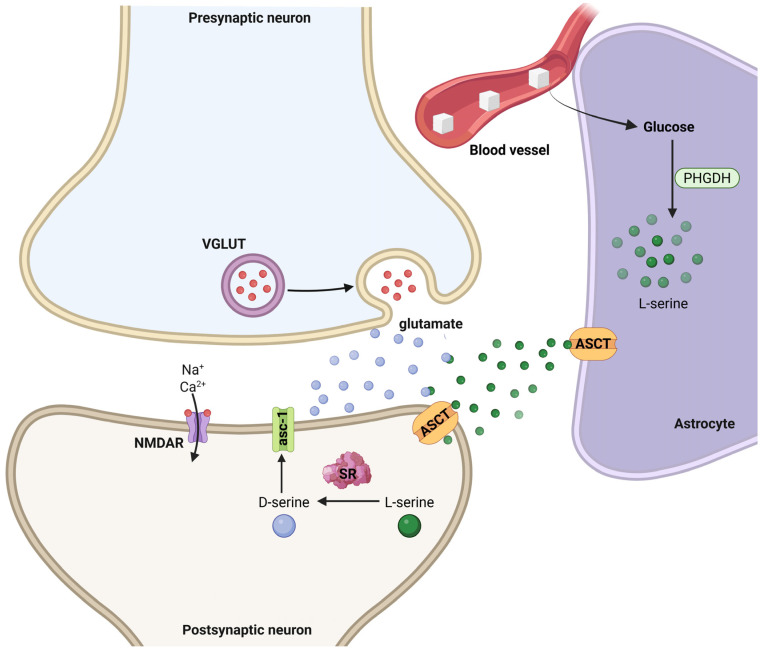
Astrocyte-neuron interactions mediating D-serine synthesis and NMDA receptor modulation at the tripartite synapse. Glucose from the blood vessel is taken up by astrocytes and metabolized via phosphoglycerate dehydrogenase (PHGDH) to produce L-serine. L-serine is exported to neurons via alanine–serine–cysteine transporters (ASCT), where it is converted to D-serine by serine racemase (SR) in the postsynaptic compartment. D-serine acts as a co-agonist at N-methyl-D-aspartate receptors (NMDARs), which are also activated by glutamate released from presynaptic terminals through vesicular glutamate transporters (VGLUTs). NMDAR activation permits Na^+^ and Ca^2+^ influx, modulating synaptic plasticity. Created in BioRender by Elisa Taddei and Luz A. Martinez Nuncio. (2025) https://biorender.com/illustrations (accessed on 21 September 2025).

Fibrous astrocytes are elongated, stellate cells approximately 20 μm in diameter, characterized by a transparent cytoplasm densely populated with gliofilaments. These astrocytes are predominantly located in the white matter and display 50–60 long, relatively unbranched processes that extend from the cell body. The processes may reach several hundred micrometers and typically terminate freely, or form flattened expansions that contact capillaries. The terminal structures, known as perivascular endfeet or “suction cups,” facilitate the formation of a continuous and homogeneous vascular covering in conjunction with the endfeet of adjacent astrocytes. Vascular pericytes and the basal lamina are interposed between the astrocytic endfeet and capillary endothelium, contributing to blood–brain barrier (BBB) structure and function [56] (Figure 5).

In specific contexts, fibrous astrocyte processes encircle the nodes of Ranvier as perinodal expansions, forming a sheath between contiguous myelinated axon segments. These perinodal specializations are crucial for meeting the increased metabolic requirements associated with ionic fluxes during prolonged action potential propagation and ATP-dependent ion transporter activity. To satisfy these energetic demands, astrocytes provide glucose transport to nodal regions, a process driven by glycogenolysis from internal stores [33,34]. As previously mentioned, astrocytic extensions that reach the pia mater or ependymal lining contribute to glia limitans formation and metabolic interactions with ependymal cells, potentially serving as sites for metabolic exchange and signal integration. All astrocytic terminals, whether from fibrous or protoplasmic astrocytes, possess a dense array of actin microfilaments, intermediate filaments, and scaffolding proteins. These cytoskeletal components are essential for the dynamic remodeling of astrocytic processes, enabling retraction and expansion of terminal structures that participate in synaptic regulation and plasticity. Moreover, astrocytic terminals are connected via connexons, creating a functional syncytium that allows coordinated intercellular communication, regulating ionic and metabolic conditions, and supporting brain homeostasis [57]. Fibrous astrocytes contribute to gliotransmission by releasing glutamate, ATP, and D-serine, which modulate synaptic activity at the tripartite synapse. They express vesicular glutamate transporters (vGluts), enabling regulated glutamate release, while D-serine synthesis via the serine shuttle modulates NMDA receptor activity and synaptic plasticity. These functions are fundamental for maintaining network excitability and supporting neuronal signaling. Astrocytic network communication also reflects Golgi’s syncytial concept of the CNS, complementing Ramón y Cajal’s neuronal theory, by enabling integrated modulation of excitability across neuronal populations.

## 6. Protoplasmic Astrocytes

Protoplasmic astrocytes constitute the predominant glial population in the gray matter of the central nervous system. These cells display a circular or polygonal soma, typically 20–25 μm in diameter. Small, highly branched processes emerge from the soma, bifurcating over short distances and forming a complex, stochastic arborization consistent with fractal geometry models of glial architecture [58,59]. This intricate morphology enables protoplasmic astrocytes to interact across extensive neuropil regions, adopting shapes influenced by neighboring neurons, glial cells, and vascular structures.

At the distal termini of their processes, protoplasmic astrocytes form specialized structures termed astrocytic endfeet, each oriented toward specific anatomical targets. Pial endfeet, periependymal endfeet, and perivascular endfeet are shared with fibrous astrocytes, contributing to glia limitans formation and blood–brain barrier maintenance. Conversely, perisynaptic endfeet are unique to protoplasmic astrocytes and encapsulate neuronal synapses, playing a central role in regulating synaptic transmission, plasticity, and gliotransmission [57]. Additionally, protoplasmic astrocytes form interglial endfeet connecting with adjacent astrocytes and other glial cells, as well as neuronal endfeet establishing direct neuron-glia contacts, features absent in fibrous astrocytes.

**Figure 6:**
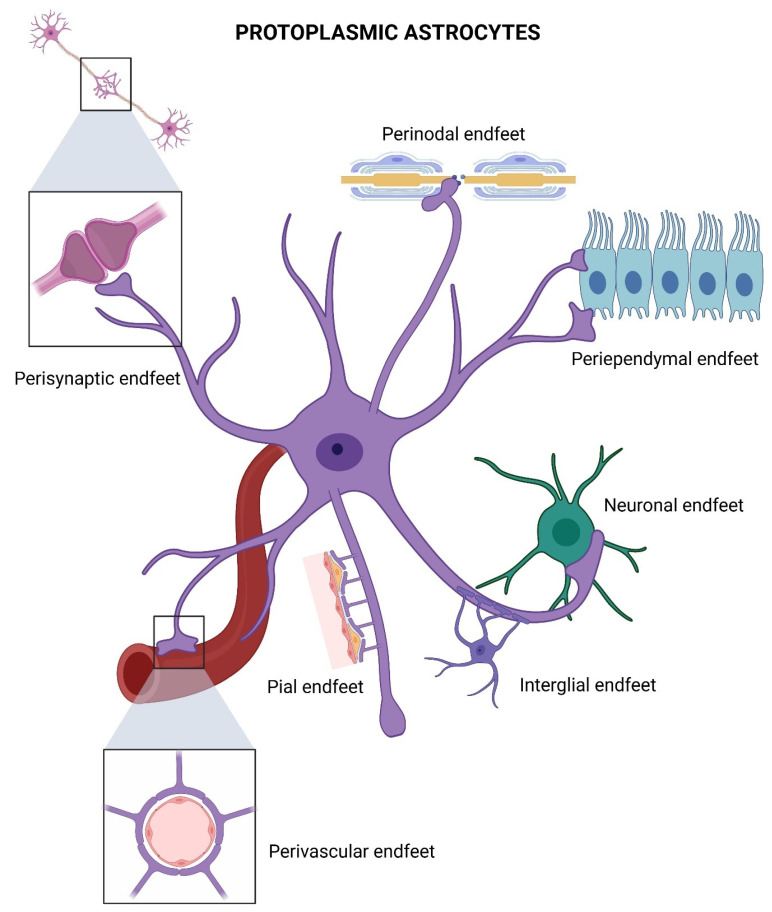
Specialized endfeet of a protoplasmic astrocyte. Perisynaptic endfeet, forming astrocytic sheaths around synapses to regulate neurotransmitter uptake and synaptic transmission. Perivascular endfeet, ensheathing blood vessels. Perinodal endfeet situated around nodes of Ranvier. Periependymal endfeet, contacting the ventricular ependymal cells to maintain barrier integrity. Interglial and neuronal endfeet, mediating astrocyte coupling. Created in BioRender by Elisa Taddei and Luz A. Martinez Nuncio. (2025) https://biorender.com/illustrations (accessed on 21 September 2025).

Protoplasmic astrocytes express vesicular glutamate transporters (vGluts), enabling the regulated release of glutamate at the tripartite synapse. Furthermore, they synthesize D-serine via the serine shuttle, modulating NMDA receptor activity and contributing to synaptic plasticity. These cells also participate in neurovascular coupling, sensing neuronal activity and coordinating vascular responses, thus integrating metabolic and ignaling support to neurons (Figure 6).

## 7. Kv10.1-Type Potassium Channels and Ion Homeostasis in Astrocytes

Astrocytes are essential for maintaining extracellular ion homeostasis in the central nervous system, particularly by regulating potassium (K^+^) concentrations. This function is mediated by a complex array of astrocytic K^+^ channels, including Kv10.1 (M10.1), Kir4.1, and two-pore domain K^+^ channels, as well as gap junction networks enabling intercellular communication [60,61,62]. Astrocytes exhibit a more negative resting membrane potential −65 mV), reflecting the combined activity of these channels. Kv10.1 channels, members of the delayed rectifier family, operate at potentials below −85 mV and facilitate K^+^ uptake from interstitial and perisynaptic regions, particularly during periods of high neuronal activity [62]. Gap junctions allow the redistribution of excess K^+^ to neighboring astrocytes, earning them the role of “spatial K^+^ buffers” [63]. Astrocytic depolarization induced by elevated extracellular K^+^ triggers glycogenolysis and glucose release to meet increased metabolic demands, notably at nodes of Ranvier. This response is critical for both myelinated and unmyelinated axons and highlights the specialized role of synantocytes. Excess extracellular K^+^, if unregulated, can disrupt neurotransmitter release, extracellular fluid volume, glucose metabolism, and neuronal excitability, emphasizing astrocytes’ pivotal role in ionic homeostasis [64]. Neuron-astrocyte communication occurs primarily through non-synaptic pathways within narrow extracellular spaces (20–30 nm), mediated by concentration gradients, electrochemical pressures, ion channels, and cell adhesion molecules (CAMs) [63,65]. Resting extracellular K^+^ is ~3 mM and rises during neuronal firing, reaching 10–12 mM in sustained activity; levels exceeding this range, as in epilepsy, can be pathophysiologically harmful [66].

**Figure 7:**
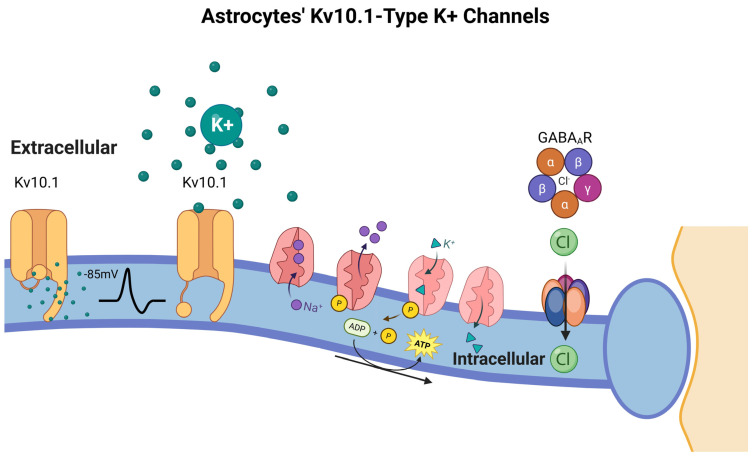
Astrocytes’ Kv10.1-Type K^+^ Channels. Kv10.1 channels, which are active below the astrocytic resting membrane potential (~−85 mV), facilitate K^+^ uptake from the extracellular space, especially during neuronal activity-induced K^+^ elevation. This K^+^ is redistributed through astrocytic gap junctions, supporting the role of astrocytes as spatial buffers. Concurrently, Na^+^/K^+^-ATPase pumps exchange intracellular Na^+^ for extracellular K^+^ to maintain ionic gradients, while Cl^−^ concentrations are regulated by GABA_A_ receptors and passive membrane properties. These mechanisms collectively preserve the extracellular ionic environment, modulate neurotransmission, and support astrocyte metabolic responses, including glycogenolysis triggered by depolarization. Dysregulation of Kv10.1 and related channels has been implicated in seizure susceptibility and other neuropathologies. Created in BioRender by Elisa Taddei and Luz A. Martinez Nuncio. (2025) https://biorender.com/illustrations (accessed on 21 September 2025).

Beyond K^+^ transport, astrocytes maintain cytoplasmic ion balance via Na^+^/K^+^-ATPase pumps, which exchange intracellular Na^+^ for extracellular K^+^, and indirectly regulate Cl^−^ homeostasis through Cl^−^ co-transporters and osmotic balance. GABA-A and glycine receptor-mediated Cl^−^ flux follows Gibbs-Donnan equilibrium principles [67]. Mutations in genes encoding M-type K^+^ channels are associated with certain epilepsies, often in combination with other clinical features [68,69]. Astrocytes also contribute to synaptic modulation through gliotransmission, releasing glutamate via vesicular transporters (vGluts) and synthesizing D-serine via the serine shuttle, which modulates NMDA receptor activity and synaptic plasticity [70,71,72,73,74]. Through these mechanisms, astrocytes integrate metabolic, ionic, and signaling support to neurons, while their gap junction networks and vascular interactions facilitate neurovascular coupling, ensuring proper neuronal function and network homeostasis (Figure 7).

## 8. Molecular and Functional Aspects of Astroglia

Astrocytes are dynamic regulators of synaptic and neuronal activity, releasing signaling molecules via membrane transporters and receptors and responding to neurotransmitters such as glutamate, GABA, and biogenic amines (serotonin, dopamine, norepinephrine) [75]. Regarding glutamate, astrocytes perform a dual function: they release glutamate through non-vesicular mechanisms, such as reverse transport, hemichannels, and vesicular exocytosis mediated by specific vesicular glutamate transporters (vGluts), while simultaneously removing excess extracellular glutamate via high-affinity excitatory amino acid transporters (EAATs) [70,72]. This bidirectional regulation maintains a delicate balance between synaptic modulation and neuroprotection, preventing excitotoxicity during intense neuronal activity.

**Figure 8:**
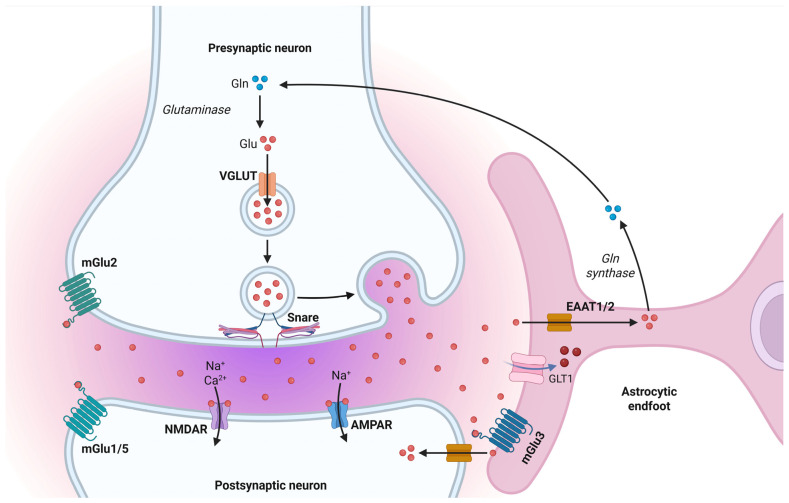
The glutamate–glutamine cycle and astrocytic regulation of excitatory neurotransmission. Glutamate (Glu) released from the presynaptic neuron is rapidly cleared from the synaptic cleft by astrocytic excitatory amino acid transporters (EAAT1/2), assisted by glutamate transporter-1 (GLT1). Once internalized, glutamate is converted into glutamine (Gln) by glutamine synthetase. Glutamine is then shuttled back to the presynaptic terminal, where glutaminase reconverts it into glutamate for vesicular packaging via the vesicular glutamate transporter (VGLUT). Postsynaptic neurons detect glutamate through ionotropic receptors (AMPAR, NMDAR) and metabotropic receptors (mGlu1/5), while astrocytes also express mGlu3 receptors to monitor and respond to synaptic glutamate dynamics Created in BioRender by Elisa Taddei and Luz A. Martinez Nuncio. (2025) https://biorender.com/illustrations (accessed on 21 September 2025).

Within milliseconds after synaptic release, astrocytes absorb glutamate at perisynaptic processes. Glutamate is then converted into glutamine, by glutamine synthetase and shuttled back to presynaptic terminals, completing the glutamate-glutamine cycle essential for synaptic transmission [76]. Astrocytes express both ionotropic (NMDA, AMPA, kainate) and metabotropic glutamate receptors (mGluRs), allowing them to sense extracellular glutamate fluctuations and trigger intracellular signaling cascades. Activation of ionotropic receptors induces membrane depolarization and facilitates extracellular K^+^ regulation through temporal reuptake mechanisms, while metabotropic receptors modulate intracellular calcium and gliotransmission [74]. Astrocytes also synthesize D-serine via the serine shuttle, releasing it to modulate NMDA receptor activity at excitatory synapses, thus directly influencing synaptic plasticity and long-term potentiation. Beyond individual synapses, astrocytes communicate through gap junctions, forming networks that enable intercellular ion and metabolite redistribution, supporting Golgi’s syncytial concept and complementing Ramón y Cajal’s neuronal doctrine [63,64]. These networks allow coordinated gliotransmission, modulate synaptic excitability across populations of neurons, and integrate metabolic and ionic support, establishing a functional tripartite synapse at each astrocyte-neuron interface. Furthermore, astrocytic endfeet interact with vascular structures, facilitating neurovascular coupling and the delivery of energy substrates to active neurons. This integration ensures proper ionic, metabolic, and signaling homeostasis throughout neural networks, highlighting the critical role of astrocytes in both physiological and pathological conditions (Figure 8).

**Figure 9:**
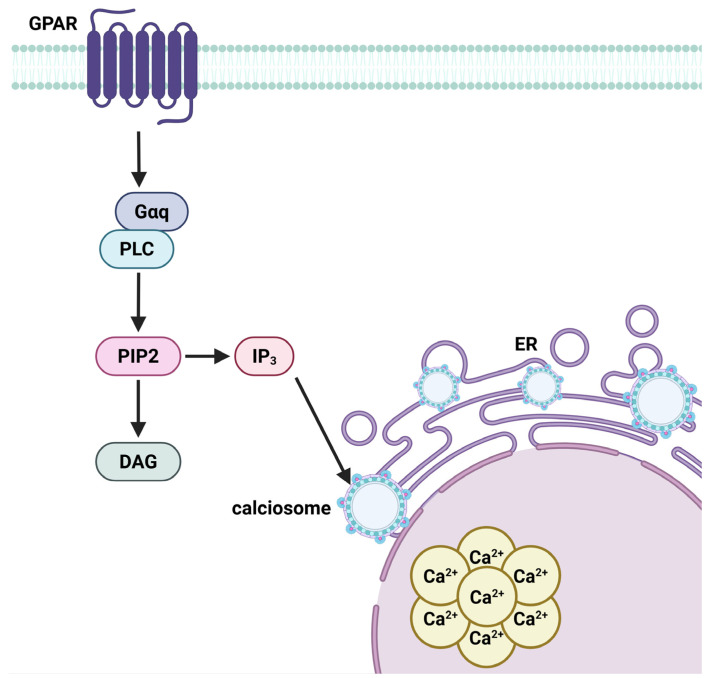
G-protein–coupled receptor (GPCR)-mediated calcium signaling cascade in astrocytes. Upon activation by extracellular glutamate, GPCRs stimulate phospholipase C (PLC), which hydrolyzes phosphatidylinositol 4,5-bisphosphate (PIP2) into diacylglycerol (DAG) and inositol 1,4,5-trisphosphate (IP_3_). IP_3_ binds to receptors on the endoplasmic reticulum (ER), releasing calcium ions (Ca^2+^) into the cytoplasm from astrocytic calcium receptors known as calciosomes. The resultant intracellular Ca^2+^ elevation initiates key astrocytic functions, including glycogenolysis and mitochondrial activation, promoting glutamate synthesis. This calcium signaling underpins astrocyte-mediated gliotransmission, metabolic support, and synchronized network activity via intercellular Ca^2+^ wave propagation. Created in BioRender by Elisa Taddei and Luz A. Martinez Nuncio. (2025) https://biorender.com/illustrations (accessed on 21 September 2025).

Simultaneously, metabotropic receptors associated with G-proteins activate intracellular pathways involving phospholipase C (PLC) or cyclic AMP (cAMP). When glutamate binds to specific G protein-coupled metabotropic receptors (mGluRs) on astrocytes, the Gαq subunit activates phospholipase C (PLC), an intracellular enzyme that catalyzes the breakdown of phosphatidylinositol bisphosphate (PIP_2_) into the second messengers diacylglycerol (DAG) and inositol trisphosphate (IP_3_) [77]. IP_3_ subsequently attaches to receptors on the endoplasmic reticulum, referred to as calciosomes in astrocytes, initiating the release of Ca^2+^ into the cytosol (Figure 9). The elevation of intracellular calcium triggers metabolic reactions, including glycogenolysis and mitochondrial activity, leading to the synthesis of α-ketoglutarate and subsequent creation of glutamate [78].

Calcium signaling in astrocytes is a defining characteristic of their integrative activity. Intracellular Ca^2+^ waves can disseminate via astrocytic networks via gap junctions, creating a functioning syncytium. These waves can vary from quick occurrences (tens of milliseconds) to extended events (up to several minutes), with their spatial dynamics influenced by the intricate geometry of astrocytic processes. Compartmentalized Ca^2+^ microdomains (50–200 nm) facilitate localized transmission, while wider waves synchronize astrocyte responses across extensive brain areas [79]. This signaling is crucial for synaptic regulation, gliotransmitter release, and neurovascular coupling (Figure 9).

In summary, astrocytes actively integrate synaptic impulses via receptor-mediated and calcium-dependent processes. Their ability to detect, react to, and regulate neuronal activity underscores their crucial role in central nervous system function, extending far beyond mere passive support.

## 9. Astrocyte Morphology and Its Role in Neurological Disorders

Astrocytes display remarkable morphological complexity, with star-shaped somata and extensively ramified processes that allow interactions with neurons, synapses, and blood vessels. These processes form perisynaptic astrocytic processes (PAPs) that envelop synapses, and astrocytic endfeet that contact capillaries, integrating astrocytes into the tripartite synapse and the neurovascular unit [80]. Peripheral glial cells, such as Müller cells or Schwann cells, are mentioned only when directly relevant. The astrocytic cytoskeleton, primarily composed of gliofilaments including GFAP, along with microtubules and actin microfilaments, maintains morphology and modulates astrocyte dynamics during synaptic plasticity or reactive gliosis [81]. Alterations in cytoskeletal components are markers of astrocytic reactivity in diseases like epilepsy or neurodegeneration [38]. Astrocytic membranes express receptors and transporters for neurotransmitters (e.g., glutamate, GABA, dopamine, serotonin) and initiate intracellular signaling cascades via G-protein-coupled receptors (GPCRs), including mGluRs, leading to regulation of IP_3_, cAMP, and Ca^2+^ signaling [82]. These pathways govern gliotransmitter release and gene transcription. Astrocytes regulate extracellular glutamate via excitatory amino acid transporters (EAAT1/GLAST and EAAT2/GLT-1), preventing excitotoxicity [83]. Potassium buffering, mediated by Kir4.1 and other potassium channels, stabilizes neuronal excitability [60]. Aquaporin-4 (AQP4), in endfeet facilitates water and solute exchange across the blood–brain barrier [84]. Astrocytic dysfunction contributes to neurological disorders by impairing glutamate uptake, potassium buffering, calcium signaling, and gliotransmission. In epilepsy, these defects exacerbate hyperexcitability [62]. In Alzheimer’s and Parkinson’s diseases, astrocytes promote oxidative stress, synaptic loss, and inflammatory signaling. Reactive astrogliosis, marked by hypertrophy and increased GFAP, may be neuroprotective or neurotoxic depending on context [85,86]. Astrocytes are key mediators of gliotransmission, releasing neurotransmitters such as D-serine, ATP, and glutamate, which modulate NMDA receptors and synaptic plasticity. They also participate in vesicular glutamate transport, expressing vGlut isoforms, and in the serine shuttle, producing D-serine to regulate excitatory synapses [72,73,74,80]. Network communication among astrocytes via gap junctions reflects Golgi’s syncytial concept, complementing neuronal circuits described by Ramón y Cajal. Astrocytes secrete neurotrophic factors, including BDNF and GDNF, supporting neuronal survival, synaptic remodeling, and memory formation [3,73,87,88,89,90]. NG2 glia, potentially differentiating into astrocytes, may enhance plasticity under glutamatergic and dopaminergic co-activation, promoting gliotransmitter release and synapse formation [25].

## 10. Conclusions

**Figure 10:**
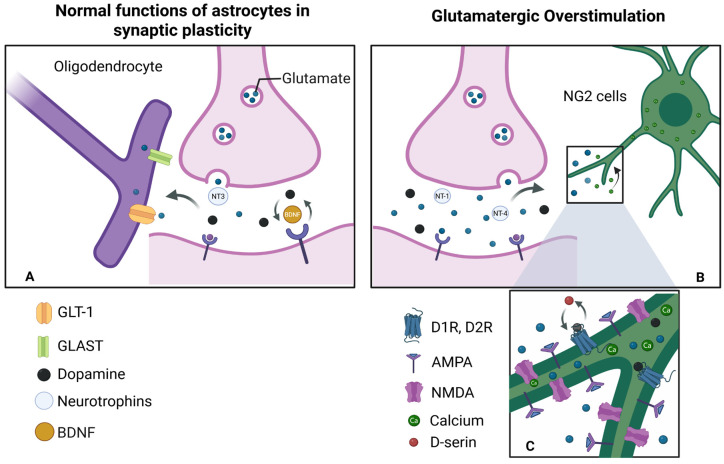
Models of stimulation and overstimulation: (**A**) During physiological stimulation of an active synapse, glutamate is released at normal levels. In this context, NG2 cells and nearby oligodendrocytes moderately express α-amino-3-hydroxy-5-methyl-4-isoxazolepropionic acid (AMPA) and N-methyl-D-aspartate (NMDA) receptors and produce trophic factors such as BDNF at basal levels. In response to this activation, cells secrete neurotrophic molecules such as BDNF, which regulate the formation, maintenance, and elimination within synapses, generating plasticity. (**B**) Overstimulation by glutamate: When glutamate is released excessively, synapses become hyperactive. Therefore, NG2 cells are unable to absorb all of the available glutamate, thus generating an intense and sustained activation of AMPA and NMDA receptors. (**C**) Signal integration: The involvement of these receptors generates an increase in intracellular calcium intake, triggering functional alterations. This is also true for the activation of both pathways: glutamatergic (NMDA, AMPA) and dopaminergic (D1, D2). This releases gliotransmitters such as D-serine, which enhance the activity of dopaminergic receptors and promotes processes such as long-term potentiation (LTP). Created in BioRender by Elisa Taddei and Luz A. Martinez Nuncio. (2025) https://biorender.com/illustrations/ (accessed on 21 September 2025).

This review emphasizes the central role of astrocytes in synaptic plasticity, neuronal homeostasis, and metabolic support, while maintaining the relevance of the neurocentric paradigm. The integrated neuron–astrocyte–capillary unit is critical for the proper functioning of the central nervous system (CNS), and astrocytic dysfunction has been associated with excitotoxicity, oxidative stress, impaired gliotransmission, and neurodegeneration. Key mechanisms underlying these processes include GPCR-mediated signaling, particularly through mGluRs, cAMP, and PKA pathways [45,91]; calcium-dependent modulation of perisynaptic astrocytes and gliotransmitter release [80]; and the regulation of potassium and glutamate homeostasis via Kir4.1 and EAATs [60,83]. Additionally, the synthesis of D-serine through the serine shuttle plays a pivotal role in NMDA receptor modulation [72,80], while astrocytic syncytia, connected through gap junctions, facilitate network communication and metabolic coupling. Collectively, these mechanisms highlight that astrocytic remodeling and signaling are essential for synaptic plasticity, memory consolidation, and the maintenance of neural circuits. Nevertheless, further research is needed to better understand the specific contributions and interplay of astrocytes, oligodendrocytes, and microglia in both healthy and diseased states [92] (Figure 10).

**Figure 11:**
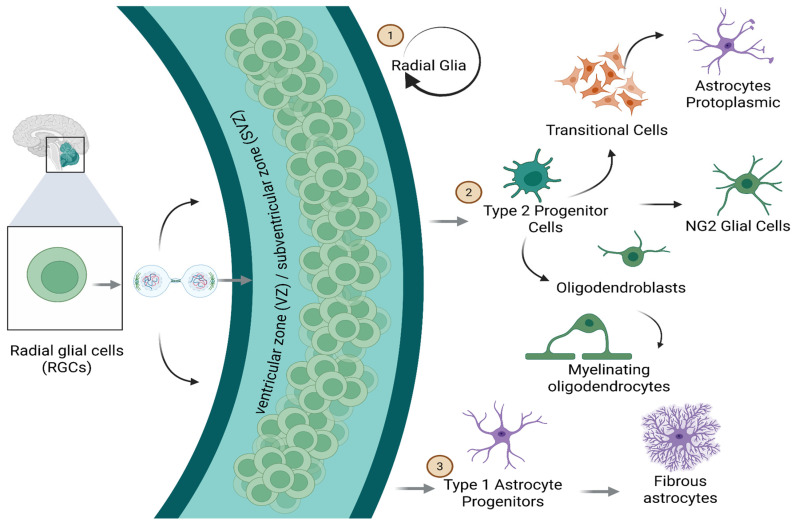
Cellular Lineages. Radial glial cells located in the ventricular/subventricular zone (VZ/SVZ) act as stem cells at the neuronal level, giving rise to cell types of different lineages. Type 2 progenitor cells and transitional cells act as intermediates; radical glial cells differentiate into protoplasmic astrocytes, NG2 glial cells, oligodendroblasts (which mature into myelinating oligodendrocytes), and type 1 astrocyte progenitors, which give rise to fibrous astrocytes. Created in BioRender by Elisa Taddei and Luz A. Martinez Nuncio. (2025) https://biorender.com/illustrations (accessed on 21 September 2025).

NG2 cells are partially undifferentiated glial progenitors originating from embryonic stem cells or radial glia [38]. These progenitors maintain mitotic capability for a finite duration, during which they proliferate and differentiate into diverse glial lineages. They pursue three primary differentiation trajectories: (1) maintenance of radial glial identity, (2) differentiation into type 1 astrocytic precursors (which develop into fibrous astrocytes), and (3) advancement into type 2 or O-2A progenitor cells. Type 2 progenitors may subsequently differentiate into transitional forms, ultimately evolving into postmitotic protoplasmic astrocytes. Alternatively, they may differentiate into oligodendroblasts, which can develop into either myelinating or satellite oligodendrocytes, or into the NG2 lineage, which retains mitotic activity until maturity and potentially beyond, across the CNS (Figure 11).

- **Perspectives**

The expanding understanding of astrocytes and other glial cells in synaptic modulation, ionic homeostasis, gliotransmission, and inflammatory responses highlights their central role in brain function, moving beyond the traditional neurocentric paradigm. Although neuronal plasticity remains essential for higher-order functions such as memory, learning, and post-injury recovery, it is increasingly evident that these processes critically depend on the trophic, structural, and metabolic interactions with glial cells. Astrocytes, in particular, actively shape the synaptic environment through glutamate uptake, release of gliotransmitters including D-serine, and secretion of neurotrophic factors such as BDNF and GDNF. This bidirectional interaction suggests that network remodeling involves not only neuronal plasticity but also astrocytic and glial plasticity, a concept that warrants further investigation. Recent findings on NG2 glial cells, which may differentiate into oligodendrocytes and possibly astrocytes, reveal a progenitor pool that remains functionally active in the adult brain. This raises important questions regarding their role in adult neurogenesis, post-ischemic repair, synaptic reorganization, and long-term potentiation (LTP), particularly in concert with glutamatergic and dopaminergic signaling and gliotransmitter release. The study of astrocytic dysfunction in pathological contexts, including epilepsy, neurodegenerative diseases such as Alzheimer’s and Parkinson’s, and affective disorders, offers new avenues for identifying early biomarkers and therapeutic targets. For example, the upregulation of GFAP and other cytoskeletal proteins often precedes overt neuronal loss and may serve as an early indicator of neurodegenerative processes.

Future research should focus on:

Precisely characterizing the intracellular signaling pathways specific to each glial subtype, including calcium and cAMP-mediated mechanisms.

Clarifying the contribution of glial cells to structural and functional synaptic plasticity, emphasizing the roles of perisynaptic astrocytic processes and gliotransmission.

Exploring the regenerative potential of glial progenitors, such as NG2 cells, in models of injury, aging, and synaptic remodeling.

Developing pharmacological, nutraceutical, or gene-targeted interventions that modulate glial function in neuropsychiatric and neurodegenerative disorders.

Adopting a neuroglio-centric perspective provides a more comprehensive understanding of CNS development, learning, homeostasis, repair, and network remodeling, underscoring the integral roles of astrocytes and glial networks in health and disease.

## Data Availability

No new data were created or analyzed in this study. Data sharing is not applicable to this article.

## Abbreviations

ADP	Adenosine Diphosphate
AMPA	α-amino-3-hydroxy-5-methyl-4-isoxazolepropionic acid receptor
AP	Area Postrema
ATP	Adenosine Triphosphate
BBB	Blood–Brain Barrier
BDNF	Brain-Derived Neurotrophic Factor
Ca^2+^	Calcium ion
CAMs	Cell Adhesion Molecules
cAMP	Cyclic Adenosine Monophosphate
CNS	Central Nervous System
Cl^−^	Chloride ion
CREB1	cAMP Response Element-Binding Protein 1
DAG	Diacylglycerol
D1, D2	Dopamine Receptors type 1 and 2
EAAT1/2	Excitatory Amino Acid Transporter 1 and 2
GDNF	Glial cell line-Derived Neurotrophic Factor
GFAP	Glial Fibrillary Acidic Protein
GFAPα, β, μ, Δ/k, Δ135, Δ164, Δexon6	GFAP isoforms
Glu	Glutamate
Gln	Glutamine
G6P	Glucose-6-Phosphate
IP3	Inositol 1,4,5-trisphosphate
IP3R2	IP3 Receptor Type 2
K^+^	Potassium ion
LDH	Lactate Dehydrogenase
LTP	Long-Term Potentiation
ME	Median Eminence
NADH	Nicotinamide Adenine Dinucleotide (reduced form)
Na^+^	Sodium ion
NG2	Neural/Glial antigen 2
NFs	Neurotrophic Factors
OPCs	Oligodendrocyte Precursor Cells
OVLT	Organum Vasculosum of the Lamina Terminalis
PAF	Platelet-Activating Factor
PC	Pericyte
PG	Pineal Gland
ROS	Reactive Oxygen Species
SCO	Subcommissural Organ
SFO	Subfornical Organ
SNARE	Soluble NSF Attachment Protein Receptor
SVZ	Subventricular Zone
VGLUT	Vesicular Glutamate Transporter
VZ	Ventricular Zone
VZ/SVZ	Ventricular/Subventricular Zone
